# Sex differences in the effects of repeated ketamine infusions on bone markers in patients with unipolar and bipolar depression

**DOI:** 10.1186/s13293-024-00587-2

**Published:** 2024-01-29

**Authors:** Xiaofeng Lan, Haiyan Liu, Chengyu Wang, Weicheng Li, Fan Zhang, Zhibo Hu, Xiaoyu Chen, Zerui You, Yuping Ning, Yanling Zhou

**Affiliations:** 1 Department of Child and Adolescent Psychiatry, Affiliated Brain Hospital of Guangzhou Medical University, Mingxin Road #36, Liwan District, Guangzhou, 510370 China; 2 Key Laboratory of Neurogenetics and Channelopathies of Guangdong Province and the Ministry of Education of China, Guangzhou Medical University, Guangzhou, 510370 China; 3 Guangdong Engineering Technology Research Center for Translational Medicine of Mental Disorders, Guangzhou, 510370 China; 4 Department of Psychology, The First School of Clinical Medicine, Southern Medical University, Guangzhou, 510515 China

**Keywords:** Ketamine, Depression, Bone mineral density, Bone markers, Leptin, Osteocalcin

## Abstract

**Background:**

Patients with depression, especially women, are associated with low bone mineral density (BMD). Traditional antidepressants are associated with negative effects on BMD. Few studies have examined the effect of ketamine on BMD, and it remains unclear whether there are sex differences in the effects of ketamine on BMD in patients with depression.

**Methods:**

A total of 102 patients with unipolar and bipolar depression were administered six infusions of intravenous ketamine over a 12-day period. Plasma levels of eight bone markers were examined at baseline, 24 h after the sixth infusion and again 2 weeks (Days 13 and 26).

**Results:**

Linear mixed models showed all bone markers had significant time main effect (all *p* < 0.05). Compared with baseline, the whole sample showed increased levels of leptin and osteoprotegerin at Days 13 and 26, as well as Dickkopf-related protein 1 at Day 13, and decreased levels of osteocalcin, sclerostin, osteopontin, parathyroid hormone and fibroblast growth factor 23 at Days 13 and 26 (all *p* < 0.05). Females had a higher level of leptin at Days 13 and 26, and lower levels of osteocalcin and sclerostin at Day 13 than males (all *p* < 0.05). Increases of leptin were associated with depressive symptom improvements at Day 13 and Day 26 in females (both *p* < 0.05). In males, higher baseline osteocalcin levels were associated with greater depressive symptom improvement at Day 26 (*β* = 0.414, *p* = 0.009).

**Conclusions:**

Our results suggest that repeated ketamine infusions may be associated with modulation of bone markers in patients with depression and present sex differences. Baseline osteocalcin level may be served as a predictor for the antidepressant effects of ketamine in males.

*Trial registration* Data were derived from an open label clinical trial, which was registered at Chinese Clinical Trial Registry (ChiCTR-OOC-17012239). Registered 26 May 2017. http://www.chictr.org.cn

**Supplementary Information:**

The online version contains supplementary material available at 10.1186/s13293-024-00587-2.

## Plain language summary

Depression and low bone mineral density (BMD) are epidemiologically linked and traditional antidepressants may act as a risk factor for BMD. However, it is unclear whether the novel antidepressant, ketamine, has effects on bone markers in patients with depression and whether there are sex differences on these effects. Ketamine infusions may be associated with modulation of bone markers and may exert a positive effect on BMD in patients with depression, which present sex differences. The study results may inform potential strategies for prevention of low BMD during the treatment of depression. Clinicians should be aware of the bone markers because some of them may be associated antidepressant response.

**Supplementary Information:**

The online version contains supplementary material available at 10.1186/s13293-024-00587-2.

## Highlights

- Depression is usually associated with low bone mineral density (BMD) and traditional antidepressants have negative effects on BMD.
- Ketamine, as a novel antidepressant, modulated multiple bone markers and might exert a positive effect on BMD in patients with depression, which present sex differences.
- Increases of leptin were positively associated with depressive symptom improvements in females across six ketamine infusions.
- Baseline osteocalcin level may be served as a predictor for the antidepressant effects of ketamine in males.

**Supplementary Information:**

The online version contains supplementary material available at 10.1186/s13293-024-00587-2.

## Introduction

Depression has emerged as one of the commonest and disabling psychiatric illness, which has a high lifetime prevalence in the range of 10% to 15% [1]. More recently, the ongoing spread of the COVID-19 pandemic is having a profound impact on the world’s mental health, and global rates of depression increased by a massive 25% in 2020. Attributing to the high rate of recurrence and treatment failure, depression is expected to become the second leading cause of overall disease burden by 2030 [2].

**Fig. 1:**
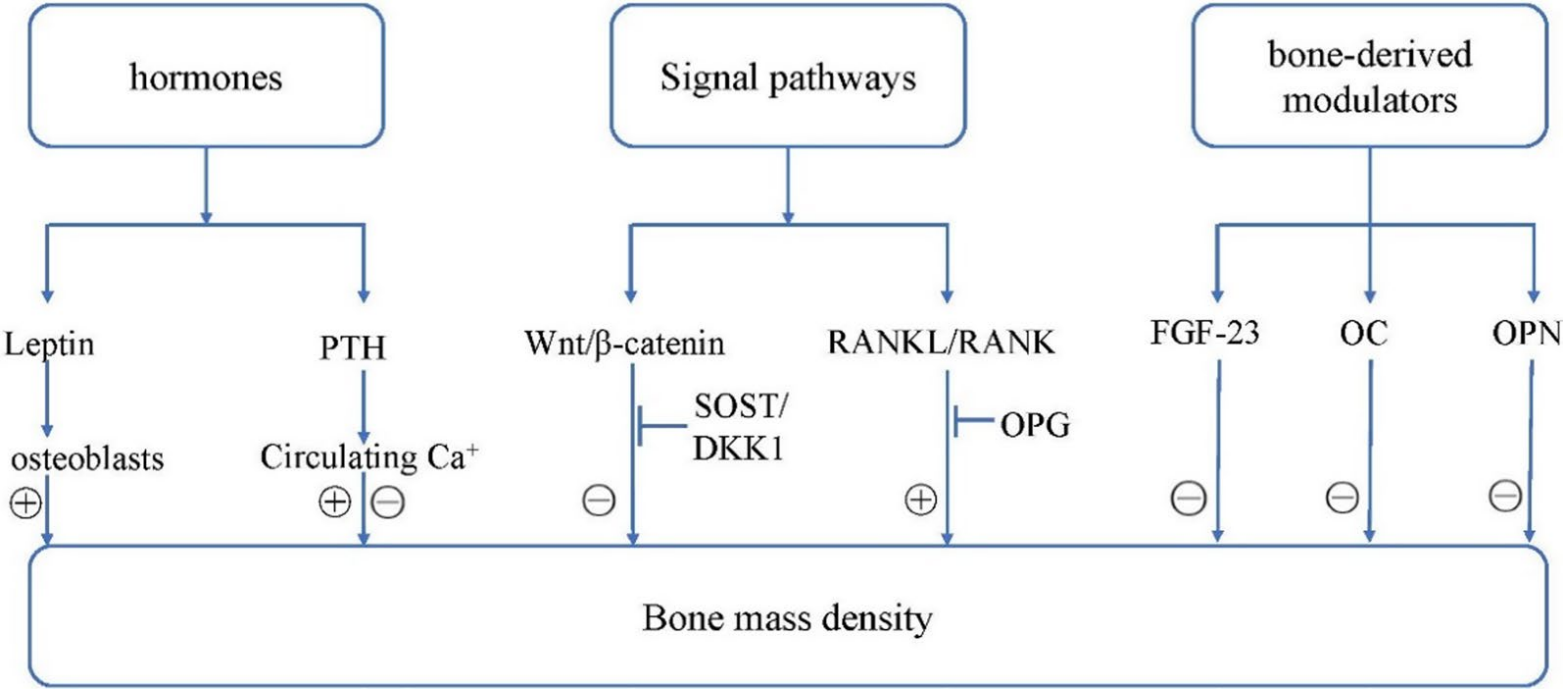
Relationships between hormones, signal pathways, bone-derived modulators and bone mass density. Leptin has a direct anabolic effect on osteoblasts while PTH exerts both anabolic and catabolic actions on bone via regulating the circulating calcium levels. DKK1 and SOST may have an adverse effect on BMD by inhibiting the Wnt/β-catenin pathway; on the opposite, OPG, as an inhibitor of RANKL/RANK pathway, may exert a beneficial effect on BMD. FGF23 regulates peripheral levels of inorganic phosphorous and 1,25-dihydroxyvitamin D_3_ and may be associated low BMD. OC is a bone turnover marker associated with decreased bone mineral density. OPN can promote bone resorption via binding osteoclasts to the mineral matrix of bones and leads to low BMD. Present study suggested that six repeated ketamine infusions increased the levels of leptin and OPG, and decreased the levels of PTH, SOST, FGF23, OC and OPN in the whole sample of patients with depression. And leptin levels had significant sex differences at each timepoint across the treatment (female > male). DKK1: Dickkopf-related protein 1; OPG: osteoprotegerin; OC: osteocalcin; OPN: osteopontin; SOST: sclerostin; PTH: parathyroid hormone; FGF23: fibroblast growth factor 23; Wnt: wingless type; RANKL: receptor activator of NF-κB ligand

Depression is usually associated with poor physical health conditions, and osteoporosis is one of them [3]. Osteoporosis is characterized by low bone mineral density (BMD) and deterioration of microarchitectural, resulting in an increased risk of fracture. Bone metabolism is affected by various factors, including hormones, local signaling pathways and bone-derived modulators, as presented in Fig. 1. Leptin and parathyroid hormone (PTH) are well-known important hormones that related to bone mass. Leptin has a direct anabolic effect on osteoblasts and it also can influence bone metabolism indirectly via many factors such as body weight, other hormones and central system [4], while PTH exerts both anabolic and catabolic actions on bone via regulating the circulating calcium levels [5]. The Wingless type (Wnt)/β-catenin pathway is an important signaling cascade in bone biology, which regulates bone development and remodeling by increasing bone formation and decreasing bone resorption [6]. Dickkopf-related protein 1 (DKK1) and sclerostin (SOST) are the most common inhibitors of the Wnt/β-catenin pathway and thus reduce BMD [6]. By contrast, in the receptor activator of NF-κB ligand (RANKL)/RANK pathway, RANKL binds to RANK as its receptor and thus promote osteoclast differentiation, eventually leading to bone loss [7]. Osteoprotegerin (OPG), acting as a decoy receptor for RANKL, can inhibit RANKL–RANK binding and thus preserves BMD [7]. Moreover, bone-derived modulators can exert regulation on the bone homeostasis. Fibroblast growth factor 23 (FGF23) is a bone-derived circulating factor that regulates peripheral levels of inorganic phosphorous and 1,25-dihydroxyvitamin D_3_ [1,25(OH)_2_D_3_]. Overexpression of FGF23 in bone is associated with increased risk of bone fractures [8]. Osteocalcin (OC), secreted exclusively by osteoblasts, is involved in bone formation and it also acts as a bone turnover marker associated with decreased bone mineral density [9]. Osteopontin (OPN) is a secreted non-collagenous protein, which was originally discovered in bone matrix and expressed in both osteoblasts and osteoclasts [10]. OPN plays a key role in bone remodeling and was considered to promote bone resorption via binding osteoclasts to the mineral matrix of bones. In short, leptin and OPG may act as the bone formation markers, while DKK1, OC, OPN, SOST and FGF-23 may as the bone resorption markers. By contrast, PTH may act either role depending on the circulating calcium levels.

Increasing studies have reported that depression has been associated with a low BMD, indicating that depression is a risk factor for osteoporosis [11, 12]. A meta-analysis of prospective studies also demonstrated that depression was associated with 26–39% increased risk of fracture [12]. Interestingly, the use of traditional antidepressants, such as tricyclic antidepressants (TCAs) and selective serotonin reuptake inhibitors (SSRIs), may have a negative effect on bone metabolism, especially in midlife women [13]. Some studies had found a significant inverse association between antidepressant use and BMD among postmenopausal women [14, 15]. In particular, SSRIs were reported to be associated with a rising risk of fractures [15]. Therefore, the development of novel antidepressants without adverse effects or even with beneficial effects for BMD is needed.

Ketamine, a nonselective N-methyl-D-aspartic acid receptor (NMDAR) antagonist, produces rapid and robust antidepressant effects in patients with major depressive disorder (MDD) or bipolar depression [16]. Meta-analyses have shown that a single ketamine IV infusion has the rapid antidepressant and antisuicidal efficacy in the treatment of unipolar and bipolar depression, but the effects could only maintain 10–12 days [16, 17]. Repeated infusion of ketamine seems to prolong the antidepressant effect of ketamine and achieve superior outcomes when compared with a single infusion [18]. Our previous publication found that 14% of patients with depression achieved an antidepressant response after the first infusion of ketamine and 68% after six infusions [19], which was consistent with prior research. These highly replicated findings suggest that ketamine is a promising antidepressant. Although ketamine showed acceptable short-term side effects such as headache, nausea, psychotomimetic and dissociative symptoms, it might have the potential risks of abuse and neurotoxicity [20]. Taking these factors into consideration, esketamine, the (S)-enantiomer of ketamine, was only approved for patients with treatment-resistant depression (TRD) or suicidality by the US Food and Drug Administration (FDA) in 2019.

In addition, it is reported that (R)-ketamine, the (R)-enantiomer of ketamine, produced greater antidepressant effect than esketamine in various rodent models [21]. An open-label pilot study also showed that (R)-ketamine exhibited fast onset and sustained antidepressant effects in TRD patients [22]. Evidences from preclinical studies indicated that (R)-ketamine could significantly attenuate the reduction of BMD in rodents with depression-like phenotypes [23, 24]. Fujita et al. conducted a randomized controlled trial (RCT) in female mice, showing that repeated (R)-ketamine administrations, rather than esketamine, can ameliorate the reduced cortical BMD and total BMD in ovariectomized mice when compared to the saline group, which occurred on 3 days after the final administration [24]. Another RCT conducted in chronic social defeat stress (CSDS) susceptible mice by Xiong et al. showed that a single injection of (R)-ketamine but not its metabolite (2R,6R)-hydroxynorketamine, significantly improved the decreased plasma ratio of OPG/RANKL and attenuated decreased BMD in CSDS susceptible mice on 3 days after the injection [23]. However, none of the preclinical trials have tested sex-different effects of ketamine or its enantiomers on BMD. Moreover, no clinical studies have examined the direct effect of ketamine or its enantiomers on BMD in patients with depression. Up to the present, clues from a clinical study suggested that ketamine may have a beneficial effect on bone metabolism in patients with depression [7]. This study reported that a single infusion of ketamine ameliorated abnormal levels of bone markers in treatment-resistant patients with MDD (*N* = 28), which occurred immediately 230 min after injection and lasted until day 3 post-infusion [7]. However, does repeated ketamine infusion produce a similar effect? Or will the effect sustain for a longer observation? Moreover, considering that osteoporosis is more common in females than males, will ketamine have a sex-different effect on bone markers level in patients with depression? Obviously, there is still a big gap needed to be filled.

Thus, we examined (a) whether bone markers are modified by repeated ketamine infusions in patients with depression and whether there are sex differences in these effects; (b) whether there is a sex difference in the association of the improvement of depressive symptoms and the changes of bone markers level across the treatment; and (c) whether baseline bone markers could predict ketamine’s antidepressant effect in different sex. We hypothesized that repeated ketamine infusions may be associated with modulation of bone markers, and the pre-treatment levels of bone markers as well as their changes would be associated with ketamine’s antidepressant effect. We expected there would be sex differences in these effects.

## Methods

Data were derived from an open label clinical trial (ChiCTR-OOC-17012239) with the aim to examine the antidepressant effect and antisuicidal effect of repeated ketamine infusions in patients with depression. The study was conducted from September 2016 to July 2018, and the first participant was enrolled on June 20, 2017. All participants were recruited in the Affiliated Brain Hospital of Guangzhou Medical University and provided written informed consent prior to entering the trial. The protocol was approved by the Clinical Research Ethics Committees of the Affiliated Brain Hospital of Guangzhou Medical University. Details of patient selection and study design have been described in our previous study [19].

### Participants

Eligible participants should meet following criteria for present study: (1) aged 18–65 years old; (2) had a diagnosis of MDD or bipolar depression without psychotic features according to the structured clinical interview for Diagnostic and Statistical Manual of Mental Disorders, fifth edition (DSM-V); (3) had a score of ≥ 17 on the 17-item Hamilton Depression Rating scale (HAMD-17); and (4) were experiencing treatment resistance defined as the absence of response to two or more classes of antidepressants with adequate dosage and treatment duration, or had a suicidal ideation confirmed by a score of ≥ 2 on Beck Scale for Suicide Ideation-part I. The exclusion criteria were as follows: (1) had a diagnosis of substance abuse or dependence, or any other severe mental disorders (such as schizophrenia and dementing disorders); (2) had any serious and unstable medical conditions; (3) was being treated with a combination of anti-inflammatory agents (i.e., nonsteroidal anti-inflammatory drugs or steroids); (4) breastfeeding women or pregnancy. If a patient was taking psychiatric medications at screening, stable dosages for more than four weeks were required prior to the trial and could be maintained throughout the infusion period.

This trial was originally designed to examine the antidepressant effect and antisuicidal effect of ketamine in patients with depression. Considering that bipolar disorder carries the highest rate of suicide of all psychiatric illnesses [25] and ketamine may be an emerging treatment for bipolar depression, patients with bipolar depression were included in present study.

### Study design and procedure

Patients received six intravenous (IV) infusions of ketamine in two weeks (3 times per week). After an overnight fast, all enrolled patients with depression received an IV infusion of ketamine at a dose of 0.5 mg/kg diluted in 0.9% saline, which was delivered over 40 min. Ketamine was administered intravenously on the 1st, 3rd, 5th, 8th, 10th and 12th days of the trial.

On the day of infusion, participants arrived in hospital in the morning after an overnight fast. Ketamine was prescribed by a psychiatrist and was prepared by a nurse in the psychiatric department. Treatment safety was monitored by a psychiatrist throughout the infusion, and an anesthesiologist were available if necessary. Temperature, blood pressure, respiratory, heart rates and mental status were periodically recorded every 10 min from one hour before the infusion to 30 min post-infusion. After each infusion, patients stayed in the treatment room for at least half hour with medical supervision until with no discomfort and then were allowed to leave with a current caregiver.

Depressive symptoms were measured by the Montgomery–Asberg Depression Rating Scale (MADRS) by clinicians at baseline, 24 h after the sixth infusion and again 2-week post-infusion (Days 0, 13, and 26).

### Biochemical analysis

Following an overnight fast, whole-blood samples were collected between 8:00 and 10:00 AM at Days 0, 13 and 26. All the obtained samples were immediately stored at 4 ℃ and then centrifuged at 3000 revolutions per minute at 4 °C for 10 min. Then the plasma was separated and stored at − 80 °C until further assayed.

Plasma concentrations of eight bone markers, including Dickkopf-related protein 1 (DKK1), leptin, osteoprotegerin (OPG), osteocalcin (OC), osteopontin (OPN), sclerostin (SOST), parathyroid hormone (PTH) and fibroblast growth factor 23 (FGF23), were detected by a Human Bone Magnetic Bead Panel (MILLIPLEX® MAP Kit, HBNMAG-51K), which was performed with the Luminex® 200™ multiplex immunoassay system according to the manufacturer’s instructions. The bone markers concentrations were calculated with MILLIPLEX® Analyst 5.1 software (EMD Millipore, Billerica, MA), using the 5-parameter logistic curve-fitting method.

### Statistical analysis

Sample size of the study should be 92 to ensure a power of 90% and an α of 0.05 by using the PASS software (NCSS, USA). Data were analyzed using SPSS statistical software for Windows, version 23, with an alpha set at two tailed p values of 0.05. We performed all analyses based on intention-to-treat (ITT) population. Missing values for the follow-up data were replaced using the last observation carried forward (LOCF) method. Participants that completed six IV infusions of ketamine with baseline blood samples were included in the final analysis.

Baseline demographic variables and clinical characteristics were compared between sex using Student’s t-test for continuous variables with a normal distribution and the Chi-square test for categorical variables. All bone markers data were natural log-transformed prior to analysis. Since it is reported that age and body mass index (BMI) have an impact on bone markers levels, covariance analysis with these variables as covariates was conducted to compare the differences in bone markers levels between sex.

Then, changes in MADRS scores and bone markers levels over time and sex differences were assessed using linear mixed models with sex and time (Days 0, 13 and 26) as factors and age and BMI as covariates. Bonferroni correction was used for post hoc comparisons of the time points examined.

Next, Pearson’s correlation analysis was applied to assessing the relationship between the improvements of depressive symptoms and the changes of bone markers levels at each time point, as well as the relationship between improvements of depressive symptoms at each time point and baseline bone markers levels. Lastly, multiple linear regression analyses were also conducted to determine the factors that were associated with improvements of depressive symptoms. In these models, the dependent variable was reductions in MADRS scores, and the independent variables were baseline levels or changes of bone markers that significantly correlated with improvements of depressive symptoms when analyzed by Pearson’s correlation, with controlling for age, BMI, current smoking, current drinking, duration of disease and dose of antidepressant. All these foregoing analyses were conducted in the total sample as well as separately for sex.

## Results

### Patient samples

In the original trial, a total of 135 patients were involved in the ketamine infusions and, 113 of them have completed six infusions and completed MADRS measurements before and after infusions. 102 of them with blood samples at baseline were included in the final analysis in the present study. At Day 13, 102 patients completed MADRS, but one patient failed to give blood sample so that only 101 blood samples were collected. At Day 26, 92 patients completed follow-up and had finished MADRS, but only 71 of them gave blood samples.

### Sex differences in baseline characteristics, depressive symptoms and bone markers

For the entire sample, 50% were female (*N* = 51), and the mean age was 34.2 ± 11.74 years. There were sex differences in some demographics between sex, including marriage, employment current smoking, duration of disease and BMI (all *p* < 0.05). Moreover, a higher proportion of MDD was observed in females (*N* = 42, 82.4%) when compared to males (*N* = 33, 64.7%) (*p* = 0.043). No significant difference was observed in MADRS score between sex (*p* > 0.05, Table 1).

**Table 1:** Demographics and clinical symptoms and level of bone markers between sex at baseline

Variables	Total (*N* = 102)	Male (*N* = 51)	Female (*N* = 51)	Statistics	
	*N* (%)	*N* (%)	*N* (%)	χ^2^	*p*
MDD	75 (73.5%)	33 (64.7%)	42 (82.4%)	4.080	0.043
Married	50 (49.0%)	18 (35.3%)	32 (62.7%)	7.689	0.006
Employed	43 (42.0%)	16 (31.4%)	27 (52.9%)	4.865	0.027
Current smoking	18 (17.6%)	15 (29.4%)	3 (5.9%)	9.724	0.002
Current drinking	3 (2.9%)	3 (5.9%)	0 (0%)	3.091	0.079
On antipsychotics	55 (53.9%)	29 (56.9%)	26 (51%)	0.355	0.551
On benzodiazepine	46 (45.1%)	21 (41.2%)	10 (19.6%)	5.607	0.018
On mood stabilizer	31 (30.4%)	21 (41.2%)	25 (49%)	0.634	0.426

	Mean ± SD	Mean ± SD	Mean ± SD	*t*	*p*
Age (year)	34.20 ± 11.74	33.45 ± 11.59	34.94 ± 11.96	0.639	0.524
Education (year)	12.47 ± 3.03	12.27 ± 3.02	12.67 ± 3.05	0.652	0.516
BMI (kg/m^2^)	22.53 ± 3.03	23.70 ± 3.73	21.35 ± 2.91	3.548	0.001
Duration of disease (months)	102.87 ± 94.40	127.04 ± 102.23	78.71 ± 79.74	2.662	0.009
Dose of antidepressant (mg/day)^a^	36.66 ± 21.95	36.82 ± 23.28	36.50 ± 20.86	0.072	0.943
MADRS score	32.14 ± 7.32	31.82 ± 7.36	32.45 ± 7.35	0.096	0.923
DKK1 (pg/ml)	6.20 ± 0.36	6.20 ± 0.33	6.21 ± 0.40	0.114	0.914
Leptin (pg/ml)	8.64 ± 1.17	8.36 ± 1.29	8.92 ± 0.97	6.238	< 0.001
OPG (pg/ml)	6.07 ± 0.30	6.08 ± 0.28	6.05 ± 0.32	0.613	0.539
OC (pg/ml)	9.91 ± 0.94	9.86 ± 0.97	9.97 ± 0.92	0.081	0.937
OPN (pg/ml)	10.2 ± 1.05	10.11 ± 1.20	10.3 ± 0.87	1.103	0.272
SOST (pg/ml)	8.01 ± 0.86	8.01 ± 0.77	8.01 ± 0.95	0.331	0.740
PTH (pg/ml)	4.42 ± 0.45	4.46 ± 0.49	4.38 ± 0.40	0.535	0.593
FGF23(pg/ml)	4.48 ± 0.43	4.57 ± 0.48	4.39 ± 0.37	1.069	0.290

All concentrations are presented as natural log-transformed

MDD: major depressive disorder; BMI: body mass index; MADRS: Montgomery–Asberg Depression Rating Scale; DKK1: Dickkopf-related protein 1; OPG: osteoprotegerin; OC: osteocalcin; OPN: osteopontin; SOST: sclerostin; PTH: parathyroid hormone; FGF23: fibroblast growth factor 23

^a^Equal dose of fluoxetine

For bone markers, females had a higher level of leptin than males (*p* < 0.001). No difference was found in other bone markers between sex after controlling for age and BMI (all *p* > 0.05, Table 1).

### Change of depressive symptoms and bone markers after six ketamine infusions

**Fig. 2:**
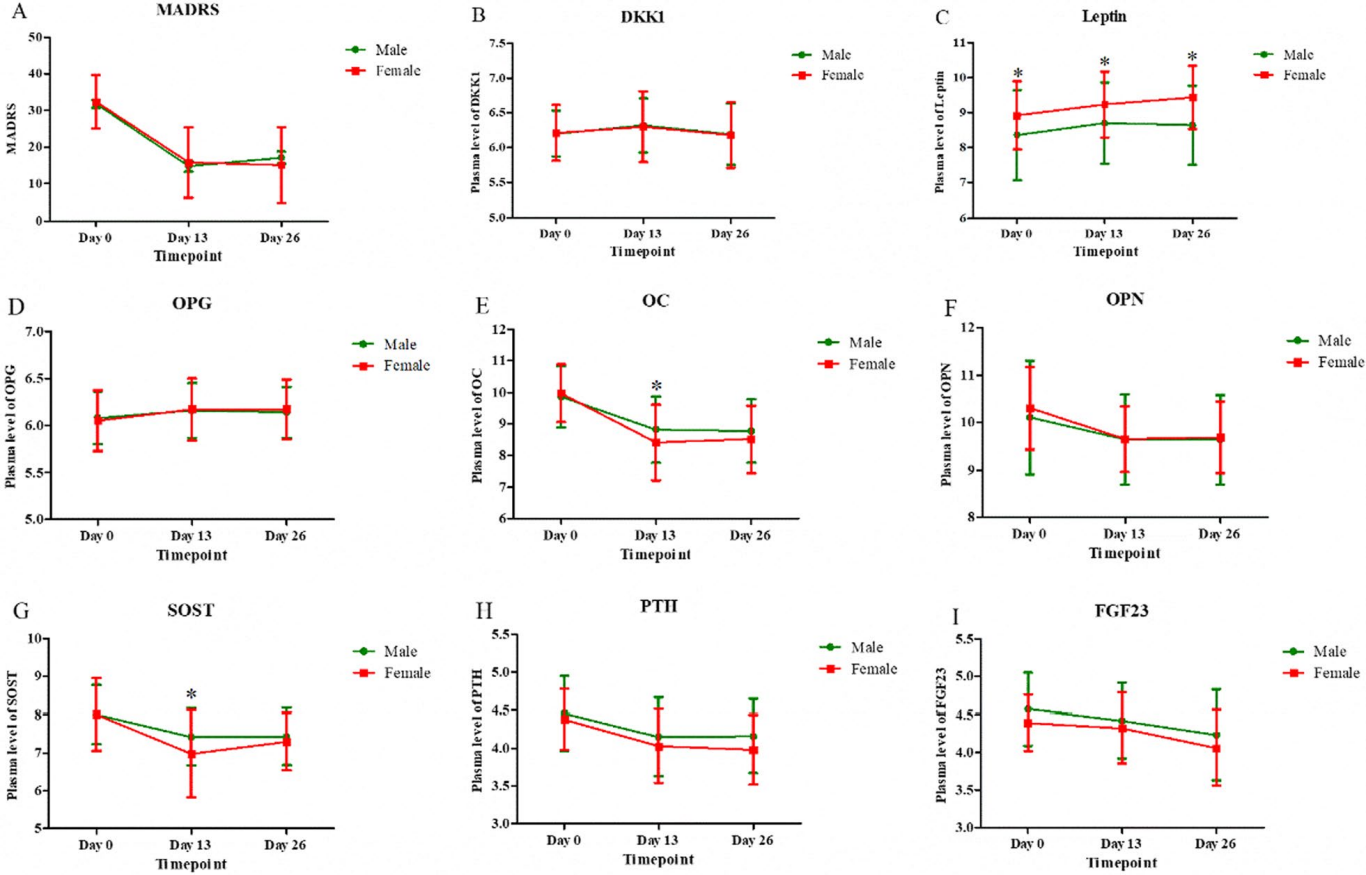
Change in depressive symptoms and bone markers in patients with unipolar and bipolar depression. *Significant difference at given time point was found between males and females (*p* < 0.05). MADRS: Montgomery–Asberg Depression Rating Scale; DKK1: Dickkopf-related protein 1; OPG: osteoprotegerin, OC: osteocalcin; OPN: osteopontin; SOST: sclerostin; PTH: parathyroid hormone; FGF23: fibroblast growth factor 23

Mean values of the MADRS scores and bone markers levels in males and females at each time point are shown in Fig. 2 and Additional file 1: Table S1.

Linear mixed models showed that MADRS scores and all bone markers levels had statistically significant time main effect (all *p* < 0.05, Table 2). For the total sample, levels of leptin and OPG at Days 13 and 26, and DKK1 at Day 13 were significantly higher than those at baseline, and levels of OC, OPN, SOST, PTH and FGF23 at Days 13 and 26 were significantly lower than those at baseline (all *p* < 0.05, Table 2).

**Table 2:** Comparison of plasma bone markers between sex

Variables	Sex-by-time interaction		Time main effect		Sex main effect	
	*F*	*p*	*F*	*p*	*F*	*p*
MARDS	1.334	0.266	178.535	< 0.001	0.118	0.732
DKK1	0.086	0.918	5.714	0.004	0.120	0.730
Leptin	4.134	0.017	34.487	< 0.001	50.361	< 0.001
OPG	1.028	0.360	12.147	< 0.001	0.002	0.965
OC	4.317	0.015	131.300	< 0.001	3.048	0.084
OPN	2.485	0.086	99.487	< 0.001	0.298	0.586
SOST	4.726	0.010	66.668	< 0.001	1.220	0.272
PTH	0.637	0.530	36.864	< 0.001	1.447	0.232
FGF23	0.490	0.613	36.980	< 0.001	0.785	0.378

BMI: body mass index; MADRS: Montgomery–Asberg Depression Rating Scale; DKK1: Dickkopf-related protein 1; OPG: osteoprotegerin; OC: osteocalcin; OPN: osteopontin; SOST: sclerostin; PTH: parathyroid hormone; FGF23: fibroblast growth factor 23

Leptin showed significant sex main effect (*F* = 50.361,  *p* < 0.001) and sex-by-time interaction (*F* = 4.134, *p* = 0.017) (Table 2 and Fig. 2). Although both males and females showed significantly higher leptin levels at Days 13 and 26 when compared to baseline, leptin levels continuously increased overtime in females but had a slight decrease in males at Day 26 (Additional file 1: Table S2 and Fig. 2). Female patients had a greater level of leptin than males at each timepoint (all *p* < 0.05, Additional file 1: Table S2 and Fig. 2).

In addition, both OC and SOST showed significant sex-by-time interaction (OC: *F* = 4.317, *p* = 0.015, SOST: *F* = 4.726, *p* = 0.010), but no sex main effect (both *p* > 0.05) (Table 2 and Fig. 2). Both males and females showed a significant decrease in OC and SOST at each timepoint when compared with baseline (Additional file 1: Table S2). Female patients had lower levels of OC and SOST than males at Day 13 but no significant difference was found between sex at Day 26 (Additional file 1: Table S2 and Fig. 2).

No significant sex-by-time interaction or sex main effect was found in other bone markers, including DKK1, OPG, OPN, PTH and FGF23 (all *p* > 0.05, Table 2).

### Association between the improvement of depressive symptoms and changes in bone markers levels

In males, the reduction of MADRS score at Day 13 was associated with the change of SOST (*r* = − 0.284, *p* = 0.044) and PTH levels according to Pearson’s correlation (*r* = − 0.395, *p* = 0.004) (Additional file 1: Table S3). But these correlations did not survive when using multivariate linear regression analysis after controlling age, BMI, current smoking, current drinking, duration of disease and dose of antidepressants (both *p* > 0.05).

**Fig. 3:**
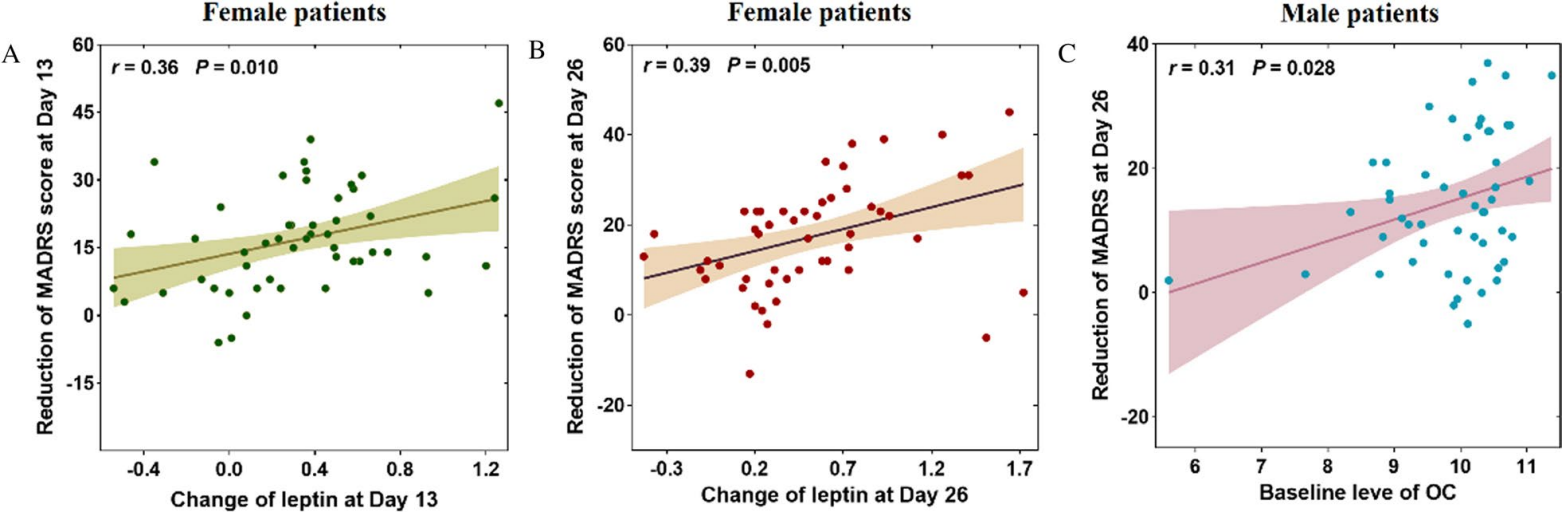
Correlation between bone markers and reductions of MADRS scores in female and male patients. MADRS: Montgomery–Asberg Depression Rating Scale; OC: osteocalcin

In females, the results of Pearson’s correlation showed that the reduction of MADRS score was related to the change of leptin at Day 13 (*r* = 0.359, *p* = 0.01; Fig. 3A) and Day 26 (*r* = 0.391, *p* = 0.005; Fig. 3B), respectively (Additional file 1: Table S3). Multiple linear regression analysis found the change of leptin was associated with the improvement of the MADRS score at Day 13 (*β* = 0.468, *p* = 0.001) and Day 26 (*β* = 0.417, *p* = 0.006), respectively.

In the total samples, no any changes of bone markers were found to be associated with reduction of MADRS (all *p* > 0.05, Additional file 1: Table S3).

### Baseline bone markers predict ketamine’s antidepressant effect

In males, baseline OC was related to the reduction of MADRS score at Day 26 (*r* = 0.307, *p* = 0.029, Additional file 1: Table S4, Fig. 3C). Multiple linear regression analysis showed baseline OC level was a predictor for the improvement of the MADRS score at Day 26 after controlling the covariates (*β* = 0.414, *p* = 0.009).

However, in females or in the total sample, no any baseline bone markers were found to be associated with the reduction of MADRS (all *p* > 0.05, Additional file 1: Table S4).

## Discussion

To the best of our knowledge, this study is the first conducted to date to examine the sex differences in the effects of repeated ketamine infusions on multiple bone markers levels and explore whether the bone markers are involved in the antidepressant effects of ketamine in patients with depression. Our study presented several significant findings. First, six infusions of ketamine associated with increased the levels of leptin and OPG, and decreased the levels of OC, OPN, SOST, PTH and FGF23 in patients with depression, which occurred immediately the day after the last infusion (Day 13) and lasted for 2-week post-infusion (Day 26). Second, females had higher leptin levels at Days 13 and 26, and lower levels of OC and SOST at Day 13 but without differences at Day 26 when compared with males. Third, increases of leptin were associated with the improvements of depressive symptoms at Days 13 and 26 in females, while higher baseline OC level predicted the improvements of depressive symptoms at Day 26 in males.

In this study, we found an increase in plasma levels of leptin and OPG, and a decrease in levels of OC, OPN, SOST, PTH and FGF23 after six ketamine infusions in adults with depression, indicating that ketamine may mediate a beneficial effect on BMD. Preclinical studies have provided clear evidence that (R)-ketamine could significantly attenuate the reduction of BMD in mice with depression-like behaviors and ovariectomized mice [23, 24]. In addition, (R)-ketamine could also improve the abnormalities in bone markers of depression-like mice after chronic social defeat stress, including increased plasma level of RANKL and decreased OPG/RANKL ratio [23]. Similarly, a clinical trial conducted by Kadriu et al. reported that MDD patients had a lower level of OPN and OPN/RANKL ratio than healthy controls, and a significant increase in the level of OPN and OPN/RANKL ratio and occurred at days 1 and 3 following a single ketamine infusion, which suggested ketamine may be able to correct abnormal bone metabolic state in MDD patients [7]. Overall, our results were consistent with previous studies and for the first time demonstrated that repeated infusions of ketamine could modulate multiple bone markers in patients with depression and this salutary effect could last for at least 2-week post-infusion, suggesting that ketamine may have a longer-term effect on bone markers. It may be of great interest that ketamine could be an ideal antidepressant in patients with depression with low BMD.

The mechanism underlying ketamine’s salutary effects on bone remains unclear. A possible mechanism may contribute to ketamine’s anti-inflammatory ability. Peripheral pro-inflammatory cytokines are involved in the pathogenesis of MDD since a subtype of MDD is known to be in a heighten pro-inflammatory state [26]. And pro-inflammatory cytokines were reported to have adverse effects on bone density [27]. Increasing evidence showed that ketamine could decrease the pro-inflammatory cytokines in patients with depression, suggesting that ketamine has a powerful anti-inflammatory effect in the treatment of depression [28, 29]. Under this scenario, ketamine may mediate bone growth by inducing inhibition of inflammation in patients with depression.

In the present study, we further explored sex differences in bone markers at baseline and during treatment. We found that females had higher baseline levels of leptin than males. Although plasma leptin levels increased both in males and females after ketamine infusion, higher leptin levels were observed in females throughout the treatment. It is well known that women have a higher level of circulating leptin than men and this sex difference is not only explained by differences in the amount of body fat, but also may be affected by their different sex steroid milieus [30]. In addition, female patients showed lower levels of OC and SOST than males immediately after the last ketamine infusion (Day 13), suggesting that females may be more sensitive to the rapid effect of ketamine on some bone markers than males. Interestingly, a preclinical study demonstrated that female rodents are more responsive to the behavioral effects of ketamine, and that this greater sensitivity is gonadal hormones-dependent [31]. Specifically, estrogen has anti-inflammation properties, and may act synergistically with ketamine to downregulate inflammation in patients with depression, thus indirectly influencing the bone markers while improving the depressive symptoms.

Furthermore, we found that the increases of leptin levels were positively associated with the improvements of depressive symptoms in females but not in males. Leptin, a peptide hormone secreted primarily by adipocytes, can affect mood and cognition via inducing structural and functional alterations in hypothalamic–pituitary–adrenal (HPA) axis, hippocampus and prefrontal cortex [32]. Preclinical research has evidenced that injection of leptin produced antidepressant activity [33], suggesting that deficiency of leptin plays a role in depression. Clinical data also showed that increase of leptin levels after antidepressant treatment was observed both in male and female patients [34]. However, only one study has examined sex differences in the relationship between the changes of leptin levels and the improvements of depressive symptoms, showing that this relationship was only observed in female patients [35], which was consistent with our results. This replicated finding suggests sex differences in leptin may be possibly influenced by their different gonadal hormone.

Interestingly, we found that baseline OC level was positively associated with depressive symptom improvement only in males following six ketamine administrations, suggesting OC may be a predictor of the antidepressant response to ketamine in male patients with depression. OC, a bone-derived protein, is recognized as a marker of bone turnover related to low BMD [9]. Apart from its impact on bone, the beneficial effects of OC on improving neurological performance were reported, such as cognition impairment, neuromotor dysfunction, and anxiety and depression [36]. However, there are inconsistent results in the relationship between OC and depressive symptoms, as well as treatment response. For example, a previous study also demonstrated that peripheral OC level was correlated with depressive severity in premenopausal women with major depression, and escitalopram (one of SSRIs) treatment increased serum OC levels [37], which might be a risk factor of osteoporosis. A recent longitudinal study conducted by Bartecku E et al. also enrolled female patients with depression and demonstrated that OC was not related to baseline depressive symptom severity, and pre-treatment OC levels did not predict response to treatment, but the decrease of OC level was associated with the improvements of depressive symptoms after 6 weeks of anti-depressive treatment [36], suggesting OC may be a candidate biomarker of antidepressant response in female patients. Nevertheless, since most studies on bone health and depression have focused on female patients, no studies have examined the relationship between OC and antidepressant response in male patients with depression. Our study for the first time reported that baseline OC may be a predictor of antidepressant response to ketamine in male patients with depression but not in females. In contrast, there was no significant association between the reduction of OC and the improvement of depressive symptoms in either sex in present study, which was inconsistent with the previous study [36]. Reasons for this discrepant result may be attribute to different follow-up period between studies. Patients in the study conducted by Bartecku E et.al had an 8-week follow-up period [36], while participants in our study only had a 26-day follow-up period. The reduction of OC level in the prior study might be greater after a longer treatment period and might be more likely to exhibit predictive effect. However, reasons for the baseline OC in sex specific effect on treatment response in present study are not yet clear and further research is needed.

Several limitations should be considered when interpreting these results. First, there was a lack of healthy control group in this study. Therefore, we could not know how the bone markers’ signatures of patients when compared to healthy volunteers’ values. In addition, it is not clear whether the relationship between MADRS scores and bone markers was due to the effect of ketamine since a control group was absent. However, it was difficult to administer ketamine to health volunteers ethically. Second, included patients were treatment-resistant or with suicidality, which might limit the generalizability of these results to patients with depression as a whole. In addition, due to the limited sample size, patients with unipolar and bipolar depression analyzed as a whole may increase heterogeneity. Third, although patients continued medications at the same stable dosages during the trial, we cannot eliminate the effects of these medications on bone markers. Fourth, we did not assess sex hormones which may potentially affect the bone markers. Further research is needed to measure the sex hormones to better illuminate the mechanism underling sex difference in bone markers during the treatment.

## Conclusion

This study showed that repeated ketamine infusions may be associated with modulation of bone markers in patients with depression, and this beneficial effect can last for at least 26 days. In addition, there are sex differences in leptin, OC and SOST levels during ketamine administrations. Increases in leptin were associated with depressive symptom improvements in female patients, while baseline OC level was predictive of antidepressant response to ketamine. Overall, our findings suggest that repeated infusions of ketamine may produce a positive and longer-term effect on BMD, and leptin and OC may be the potential biomarkers of antidepressant response to ketamine in female and male patients with depression, respectively.

### Supplementary Information

**Additional file 1: Table S1.** Mean values of the MADRS score and plasma bone marker in patients. **Table S2.** Results of comparisons of sex differences in timing and comparison with baseline using linear mixed-model analysis with Bonferroni-corrected post hoc tests. **Table S3.** Correlation between changes of bone markers and reductions of MADRS scores across ketamine treatment. Data are represented with r. **Table S4.** Correlation between baseline bone markers and reductions of MADRS scores across ketamine treatment. Data are represented with r.

## Data Availability

The datasets during and/or analyzed during the current study are available from the corresponding author on reasonable request.
