# Supplementary material for: Sex differences in the effects of repeated ketamine infusions on bone markers in patients with unipolar and bipolar depression
Source: Biol Sex Differ. 2024 Jan 29;15:12. doi: 10.1186/s13293-024-00587-2 (PMC10826032; doi:10.1186/s13293-024-00587-2)
Supplement: Supplementary file 1 — Additional file 1: Table S1. Mean values of the MADRS score and plasma bone marker in patients. Table S2. Results of comparisons of sex differences in timing and comparison with baseline using linear mixed-model analysis with Bonferroni-corrected post hoc tests. Table S3. Correlation between changes of bone markers and reductions of MADRS scores across ketamine treatment. Data are represented with r. Table S4. Correlation between baseline bone markers and reductions of MADRS scores across ketamine treatment. Data are represented with r. [file 13293_2024_587_MOESM1_ESM.docx]

Table S1. Mean values of the MADRS score and plasma bone marker in patients.

| Variables | Time | Total (N=102) | Male (N=51) | Female (N=51) |
| --- | --- | --- | --- | --- |
| MADRS | Day 0 | 32.14±7.32 | 31.82±7.36 | 32.45±7.35 |
|  | Day 13 | 15.25±10.29 | 14.75±10.98 | 15.75±9.63 |
|  | Day 26 | 16.07±11.11 | 17.08±11.78 | 15.06±10.41 |
| DKK1 | Day 0 | 6.20±0.36 | 6.20±0.33 | 6.21±0.40 |
|  | Day 13 | 6.31±0.45 | 6.32±0.39 | 6.30±0.51 |
|  | Day 26 | 6.18±0.45 | 6.19±0.44 | 6.18±0.47 |
| Leptin | Day 0 | 8.64±1.17 | 8.36±1.29 | 8.92±0.97 |
|  | Day 13 | 8.97±1.09 | 8.70±1.17 | 9.23±0.94 |
|  | Day 26 | 9.04±1.1 | 8.64±1.13 | 9.44±0.91 |
| OPG | Day 0 | 6.07±0.3 | 6.08±0.28 | 6.05±0.32 |
|  | Day 13 | 6.17±0.31 | 6.16±0.29 | 6.17±0.33 |
|  | Day 26 | 6.16±0.29 | 6.14±0.27 | 6.17±0.32 |
| OC | Day 0 | 9.91±0.94 | 9.86±0.97 | 9.97±0.92 |
|  | Day 13 | 8.61±1.13 | 8.82±1.04 | 8.41±1.19 |
|  | Day 26 | 8.64±1.04 | 8.77±1.00 | 8.51±1.07 |
| OPN | Day 0 | 10.2±1.05 | 10.11±1.20 | 10.3±0.87 |
|  | Day 13 | 9.65±0.83 | 9.64±0.95 | 9.65±0.69 |
|  | Day 26 | 9.66±0.85 | 9.64±0.94 | 9.69±0.75 |
| SOST | Day 0 | 8.01±0.86 | 8.01±0.77 | 8.01±0.95 |
|  | Day 13 | 7.2±0.99 | 7.42±0.75 | 6.98±1.15 |
|  | Day 26 | 7.37±0.75 | 7.43±0.75 | 7.30±0.75 |
| PTH | Day 0 | 4.42±0.45 | 4.46±0.49 | 4.38±0.40 |
|  | Day 13 | 4.09±0.51 | 4.15±0.52 | 4.03±0.49 |
|  | Day 26 | 4.07±0.49 | 4.16±0.49 | 3.98±0.46 |
| FGF23 | Day 0 | 4.48±0.43 | 4.57±0.48 | 4.39±0.37 |
|  | Day 13 | 4.37±0.49 | 4.42±0.50 | 4.32±0.47 |
|  | Day 26 | 4.14±0.56 | 4.23±0.60 | 4.06±0.50 |

All concentrations are presented as natural log-transformed (pg/ml)

Abbreviations: MADRS=Montgomery-Asberg Depression Rating Scale, DKK1= Dickkopf-related protein 1, OPG= Osteoprotegerin, OC= osteocalcin, OPN= osteopontin, SOST= Sclerostin, PTH= parathyroid hormone, FGF23= Fibroblast growth factor 23.

Table S2. Results of comparisons of sex differences in timing and comparison with baseline using linear mixed-model analysis with Bonferroni-corrected post hoc tests.

| Variables | Time | Comparison between sex | | Comparison with baseline | | | | | |
| --- | --- | --- | --- | --- | --- | --- | --- | --- | --- |
|  |  |  |  | Total (N=102) | | Male (N=51) | | Female (N=51) | |
|  |  | t | p^a^ | t | p^a^ | t | p^a^ | t | p^a^ |
| MADRS | Day 0 | 0.096 | 0.923 |  |  |  |  |  |  |
|  | Day 13 | 0.282 | 0.779 | 16.758 | <0.001 | 11.976 | <0.001 | 11.715 | <0.001 |
|  | Day 26 | 1.219 | 0.225 | 15.941 | <0.001 | 10.340 | <0.001 | 12.196 | <0.001 |
| DKK1 | Day 0 | 0.114 | 0.914 |  |  |  |  |  |  |
|  | Day 13 | 0.477 | 0.630 | 2.600 | 0.027 | 2.143 | 0.097 | 1.571 | 0.356 |
|  | Day 26 | 0.295 | 0.768 | 0.525 | 1.000 | 0.214 | 1.00 | 0.518 | 1.000 |
| Leptin | Day 0 | 6.238 | <0.001 |  |  |  |  |  |  |
|  | Day 13 | 6.076 | <0.001 | 6.327 | <0.001 | 4.699 | <0.001 | 4.315 | <0.001 |
|  | Day 26 | 7.651 | <0.001 | 7.769 | <0.001 | 3.863 | <0.001 | 7.192 | <0.001 |
| OPG | Day 0 | 0.613 | 0.539 |  |  |  |  |  |  |
|  | Day 13 | 0.097 | 0.929 | 4.348 | <0.001 | 2.469 | 0.044 | 3.813 | 0.001 |
|  | Day 26 | 0.403 | 0.685 | 4.000 | <0.001 | 1.875 | 0.183 | 3.844 | <0.001 |
| OC | Day 0 | 0.081 | 0.937 |  |  |  |  |  |  |
|  | Day 13 | 2.589 | 0.011 | 14.141 | <0.001 | 7.992 | <0.001 | 12.023 | <0.001 |
|  | Day 26 | 1.861 | 0.065 | 13.859 | <0.001 | 8.377 | <0.001 | 11.238 | <0.001 |
| OPN | Day 0 | 1.103 | 0.272 |  |  |  |  |  |  |
|  | Day 13 | 0.113 | 0.912 | 12.400 | <0.001 | 7.203 | <0.001 | 10.219 | <0.001 |
|  | Day 26 | 0.361 | 0.717 | 11.978 | <0.001 | 7.313 | <0.001 | 9.547 | <0.001 |
| SOST | Day 0 | 0.331 | 0.740 |  |  |  |  |  |  |
|  | Day 13 | 2.545 | 0.012 | 10.919 | <0.001 | 5.635 | <0.001 | 9.894 | <0.001 |
|  | Day 26 | 0.558 | 0.580 | 8.649 | <0.001 | 5.500 | <0.001 | 6.808 | <0.001 |
| PTH | Day 0 | 0.535 | 0.593 |  |  |  |  |  |  |
|  | Day 13 | 0.949 | 0.344 | 7.130 | <0.001 | 4.723 | <0.001 | 5.354 | <0.001 |
|  | Day 26 | 1.576 | 0.118 | 7.630 | <0.001 | 4.615 | <0.001 | 6.185 | <0.001 |
| FGF23 | Day 0 | 1.069 | 0.290 |  |  |  |  |  |  |
|  | Day 13 | 0.347 | 0.730 | 2.725 | 0.020 | 2.589 | 0.031 | 1.286 | 0.595 |
|  | Day 26 | 0.970 | 0.338 | 8.375 | <0.001 | 6.071 | <0.001 | 5.893 | <0.001 |

Abbreviations: MADRS=Montgomery-Asberg Depression Rating Scale, DKK1= Dickkopf-related protein 1, OPG= Osteoprotegerin, OC= osteocalcin, OPN= osteopontin, SOST= Sclerostin, PTH= parathyroid hormone, FGF23= Fibroblast growth factor 23.

a: controlling for age, gender and BMI.

Table S3 Correlation between changes of bone markers and reductions of MADRS scores across ketamine treatment. Data are represented with r.

| Variables | Total | | Male | | Female | | |
| --- | --- | --- | --- | --- | --- | --- | --- |
|  | Reduction of MADRS at Day 13 | Reduction of MADRS at Day 26 | Reduction of MADRS at Day 13 | Reduction of MADRS at Day 26 | Reduction of MADRS at Day 13 | Reduction of MADRS at Day 26 | |
| ΔDKK1 | -0.031  0.022  -0.075  0.061  0.071  -0.019  -0.131  -0.103 | -0.165  0.135  0.043  -0.040  0.051  -0.027  0.044  0.082 | -0.197  -0.227  -0.257  -0.180  0.037  -0.284*  -0.395*  -0.056 | -0.115  -0.101  -0.069  -0.114  -0.005  -0.155  -0.044  0.013 | 0.120  0.359*  0.140  0.234  0.103  0.155  0.103  -0.136 | -0.221  0.391*  0.135  0.051  0.157  0.109  0.150  0.132 |  |
| ΔLeptin |  |  |  |  |  |  |  |
| ΔOPG |  |  |  |  |  |  |  |
| ΔOC |  |  |  |  |  |  |  |
| ΔOPN |  |  |  |  |  |  |  |
| ΔSOST |  |  |  |  |  |  |  |
| ΔPTH |  |  |  |  |  |  |  |
| ΔFGF23 |  |  |  |  |  |  |  |

Abbreviations: MADRS=Montgomery-Asberg Depression Rating Scale, DKK1= Dickkopf-related protein 1, OPG= Osteoprotegerin, OC= osteocalcin, OPN= osteopontin, SOST= Sclerostin, PTH= parathyroid hormone, FGF23= Fibroblast growth factor 23.

Δ represents change of boner markers from baseline to each follow-up point. (Values at follow-up minus those at baseline)

*represents p<0.05.

Table S4. Correlation between baseline bone markers and reductions of MADRS scores across ketamine treatment. Data are represented with r.

| Variables | Total | | Male | | Female | |
| --- | --- | --- | --- | --- | --- | --- |
|  | Reduction of MADRS at Day 13 | Reduction of MADRS at Day 26 | Reduction of MADRS at Day 13 | Reduction of MADRS at Day 26 | Reduction of MADRS at Day 13 | Reduction of MADRS at Day 26 |
| DKK1 | 0.079  -0.021  -0.083  -0.027  0.029  0.034  -0.036  0.081 | 0.119  0.084  -0.086  0.020  0.019  0.079  -0.059  -0.037 | 0.186  0.185  -0.040  0.094  0.102  0.076  0.117  0.159 | 0.261  0.210  -0.043  0.307*  0.130  0.263  0.099  0.013 | 0.004  -0.251  -0.117  -0.137  -0.053  0.005  -0.200  -0.008 | 0.013  -0.121  -0.111  -0.266  -0.145  -0.055  -0.210  -0.046 |
| Leptin |  |  |  |  |  |  |
| OPG |  |  |  |  |  |  |
| OC |  |  |  |  |  |  |
| OPN |  |  |  |  |  |  |
| SOST |  |  |  |  |  |  |
| PTH |  |  |  |  |  |  |
| FGF23 |  |  |  |  |  |  |

Abbreviations: MADRS=Montgomery-Asberg Depression Rating Scale, DKK1= Dickkopf-related protein 1, OPG= Osteoprotegerin, OC= osteocalcin, OPN= osteopontin, SOST= Sclerostin, PTH= parathyroid hormone, FGF23= Fibroblast growth factor 23.

*represents p<0.05.
